# Preoperative Diagnosis of Abdominal Extra-Adrenal Paragangliomas with Fine-Needle Biopsy

**DOI:** 10.3390/diagnostics12081819

**Published:** 2022-07-28

**Authors:** Ilias P. Nikas, Angela Ishak, Mousa M. AlRawashdeh, Eirini Klapsinou, Athanasia Sepsa, George N. Tzimas, Dimitrios Panagiotakopoulos, Dimitrios Papaioannou, Charitini Salla

**Affiliations:** 1 School of Medicine, European University Cyprus, Nicosia 2404, Cyprus; angela.ishak.10@gmail.com (A.I.); mousa99mahmoud@gmail.com (M.M.A.); 2 Department of Cytopathology, Hygeia and Mitera Hospital, 15123 Athens, Greece; eirini_kl@yahoo.com (E.K.); charitinisalla@yahoo.com (C.S.); 3 Department of Pathology, Metropolitan Hospital, 18547 Athens, Greece; cynthia.sepsa@gmail.com; 4 Department of Hepatobiliary Surgery, Hygeia and Mitera Hospital, 15123 Athens, Greece; george.tzimas@hpb.gr; 5 Department of Gastroenterology and Hepatology, Metropolitan General Hospital, 15562 Athens, Greece; athens.gastro@gmail.com; 6 Department of Pathology, Hygeia and Mitera Hospital, 15123 Athens, Greece; dpapaioannou@hygeia.gr

**Keywords:** endoscopic ultrasound-guided fine-needle aspiration (EUS-FNA), pancreatic neuroendocrine tumor (PanNET), cytopathology, neoplasm, molecular pathology, pancreas, metastasis, cancer prognosis, immunohistochemistry, paraganglioma

## Abstract

Paragangliomas are rare, non-epithelial neuroendocrine neoplasms originating in paraganglia, for instance the adrenal medulla, or at extra-adrenal locations. The aim of this study was to review the literature regarding abdominal extra-adrenal paragangliomas diagnosed pre-operatively with fine-needle biopsy (FNA and/or FNB). The PubMed database was searched to identify such cases, using a specific algorithm and inclusion/exclusion criteria. An unpublished case from our practice was also added to the rest of the data, resulting in a total of 36 cases for analysis. Overall, 24 (67%) lesions were found in females, whereas 12 (33%) in males. Most (21/36; 58.33%) were identified around and/or within the pancreatic parenchyma. FNA and/or FNB reached or suggested a paraganglioma diagnosis in 17/36 cases (47.22%). Of the preoperative misdiagnoses, the most common was an epithelial neuroendocrine tumor (NET). Regarding follow-up, most patients were alive with no reported recurrence; however, 5/36 patients exhibited a recurrence or a widespread disease, whereas one patient died 48 months following her diagnosis. In two patients, transient hypertension was reported during the EUS-FNA procedure. In conclusion, this study showed that the preoperative diagnosis of these lesions is feasible and, while diagnostic pitfalls exist, they could significantly be avoided with the application of immunochemistry.

## 1. Introduction

Paragangliomas are rare, non-epithelial neuroendocrine neoplasms derived from the neural crest, yet their incidence has increased over recent years. Pheochromocytomas and paragangliomas (often referred together as PPGLs) are histologically similar. Notably, according to the latest WHO guidelines, pheochromocytomas are now officially considered as “intra-adrenal paragangliomas” [1,2]. PPGLs originate in structures called the paraganglia—found in close association with the autonomic nervous system—for instance in the adrenal medulla (where the term pheochromocytoma is traditionally used) or in paraganglia located at extra-adrenal locations. The latter are most often found in the head and neck area (e.g., the carotid body) or the retroperitoneum (e.g., the organ of Zuckerkandl). Head and neck paragangliomas are most likely parasympathetic, whereas retroperitoneal paragangliomas are sympathetic [1,3,4]. These neoplasms could be either functional or non-functional; the former could be detected by using a meta-iodobenzylguanidine (MIBG) scan, which results in high MIBG uptake from the lesion, or by measuring the catecholamine or their metabolites (metanephrine) levels in the plasma and/or urine of the patients [5,6]. Of interest, paragangliomas exhibit the highest genetic predisposition among all human neoplasms, and more than 40% of PPGL patients carry germline mutations; in addition, sporadic mutations are prevalent as well and, when existing alongside germline mutations, they may exhibit a synergistic effect. Examples of PPGLA mutations include the RET gene in multiple endocrine neoplasia (MEN) syndromes, the VHL gene in von Hippel–Lindau disease, the NF1 gene in Neurofibromatosis type 1, the MAX gene, or the genes of the succinate dehydrogenase complex (SDHB, SDHD, SDHA, SDHC, SDHAF2) [1,3,7,8,9].

PPGL patients could present with a clinical picture related to catecholamine hypersecretion, such as headaches, palpitation, and high blood pressure, coupled with high levels of metanephrines in their plasma or urine. In such cases, clinicians could suspect, readily identify, and subsequently manage the lesion [5,10,11]. However, this clinical presentation is often absent, and abdominal masses subsequently diagnosed as PPGLs are either identified incidentally in asymptomatic patients using modalities, such as CT or MRI, or present with non-specific symptomatology (e.g., abdominal pain). In such scenarios, clinicians do not often suspect a paraganglioma, and preoperative diagnosis largely relies on the pathologic interpretation. Preoperative diagnosis of such masses is most often performed with fine-needle aspiration (FNA) and/or fine-needle biopsy (FNB), while these procedures are typically guided with imaging, such as endoscopic ultrasound (EUS) [4,5,11]. Due to the possibility of paroxysmal hypertension, biopsies of PPGL masses are often contra-indicated, for instance when they are located in the adrenal glands; this is a main reason why pheochromocytoma biopsies are seldom performed, compared to the neoplasm’s incidence [12]. However, some authors reported that biopsy-related complications were not common in their practice, while, when they occurred, they could be controlled with the administration of adrenergic blocking [13,14].

The aim of this study was to review the literature regarding the reported cases of abdominal extra-adrenal paragangliomas diagnosed pre-operatively with FNA and/or FNB, with the goal to reveal their main demographics, diagnostic pitfalls, and clinical behavior. In addition, we also present an unpublished case of a retroperitoneal paraganglioma from our practice, diagnosed with EUS-FNB.

## 2. Materials and Methods

### 2.1. Search Strategy

The PubMed database was searched for cases of abdominal extra-adrenal paragangliomas diagnosed with FNA and/or FNB from 1 January 2000, until 26 May 2022, using the following algorithm: (paraganglioma OR pheochromocytoma) AND (FNA OR “fine-needle aspiration” OR FNB OR EUS-FNA OR EUS-FNB).

### 2.2. Study Selection

Inclusion criteria involved any studies (e.g., cohort, case series, case reports) reporting abdominal extra-adrenal paragangliomas diagnosed with FNA and/or FNB, where the final diagnosis was reached either preoperatively from the FNA/FNB material or by examining the subsequent surgical excision specimen. Exclusion criteria involved studies reporting solely PPGLs found in the adrenals (pheochromocytomas), outside the abdomen, or diagnosed with modalities other than FNA/FNB. Articles where individual data of the paraganglioma cases described could not be extracted were also excluded at a subsequent step. Three authors (I.P.N., A.I. and M.M.A.) first performed a title–abstract selection, which was followed by a full-text evaluation of all eligible articles. Any discrepancies were resolved by reaching a consensus.

### 2.3. Data Extraction

The following data regarding individual paraganglioma cases were extracted in an Excel^®^ file: first author, year of publication, gender, age, location of the mass in the abdomen, diameter of the mass assessed with endoscopy or radiology (e.g., EUS or CT), clinical presentation, biopsy type (FNA and/or FNB), preoperative diagnosis derived from the FNA and/or FNB pathologic interpretation, immunochemistry performed on the FNA or FNB material, whether or not the mass was excised with surgery, and follow-up information.

## 3. Results 

### 3.1. Literature Search

**Figure 1:**
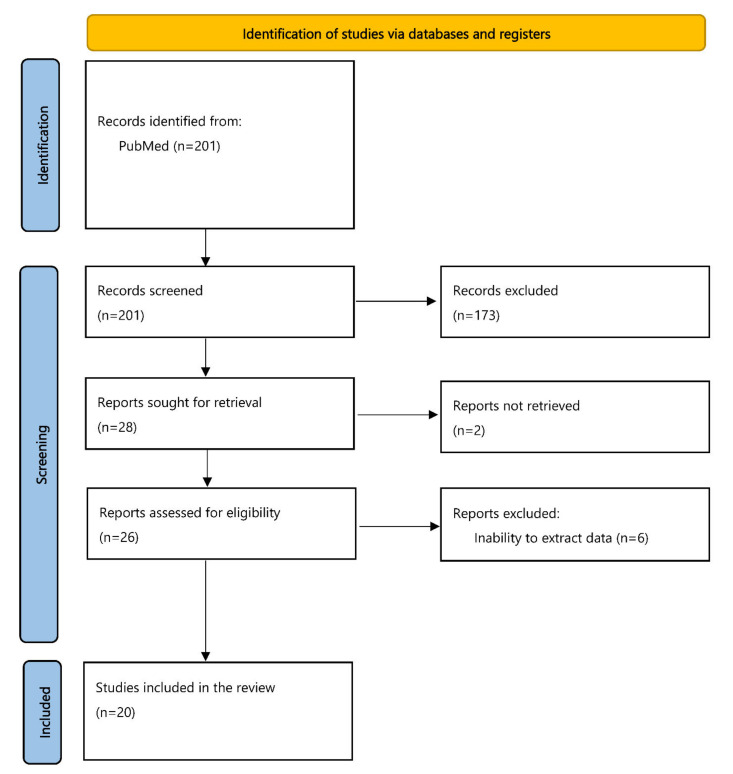
Flowchart of our study.

The flowchart of our study is shown in Figure 1. The initial search in the PubMed database identified 201 articles, which were subsequently screened in a title-abstract fashion. In total, 28 studies were eligible for full-text evaluation; of them, 2 could not retrieved, whereas 6 were excluded due to an inability to extract individual data concerning the paraganglioma cases reported. Lastly, 20 studies, reporting 35 abdominal extra-adrenal paragangliomas, were included in the review. An unpublished abdominal extra-adrenal paraganglioma case from our practice was also added to the rest of the data, resulting in a total of 36 cases for analysis.

### 3.2. Characteristics of the Included Studies

The extracted data from the 36 eligible paraganglioma cases are shown in Table 1. Overall, 24 (67%) were females, whereas 12 (33%) were males. Mean and median ages of diagnosis were 54.22 and 54.5 years, respectively. Mean age was 57.04 for females, whereas 48.58 for males. In total, 21 of the 36 abdominal paraganglioma cases (21/36; 58.33%) accessed with a fine-needle biopsy were found to be located around and/or within the pancreatic parenchyma (peripancreatic), most often in association with the pancreatic head. The diameter of the lesions ranged from 19 to 170 mm. Although a few of the cases were symptomatic at diagnosis (e.g., presence of abdominal pain), 13/36 (36%) were detected incidentally with a radiologic modality. Most lesions were retroperitoneal, while one was intraperitoneal [15]. In two patients, transient hypertension was reported during the EUS-FNA procedure [16,17]. FNA was solely used for 31/36 cases, whereas a combination of FNA and FNB for 4/36 and solely FNB for 1/36 cases, respectively. FNA and/or FNB reached or suggested a diagnosis of PPGL in 17/36 cases (47.22%). In the rest of the cases, complete pathologic examination of the surgical specimen was necessary for the final diagnosis. Of the preoperative misdiagnoses, the most common one was an epithelial neuroendocrine tumor (NET), for example a pancreatic NET (PanNET). Application of immunochemistry on cell blocks, cytology slides, or the FNB material was reported in 14/36 cases, while surgical excision of the abdominal mass in 28/36 cases. Regarding follow-up, most patients were alive with no reported recurrence; however, 5/36 patients (13.9%) were reported to have a recurrence or a widespread disease, whereas one patient (1/36; 2.8%) died 48 months following her diagnosis [18].

### 3.3. Case Report

Our case was about a 35-year-old female patient who underwent an EUS-FNB of a retroperitoneal mass, which had previously been detected incidentally. EUS revealed this mass was located in the paraduodenal area and had a diameter of 65 mm, while it was significantly vascular. Two passes with a 22G FNB needle through the duodenum were performed; cores were put directly into formalin, whereas the remaining material was used for conventional and liquid-based cytology.

**Figure 2:**
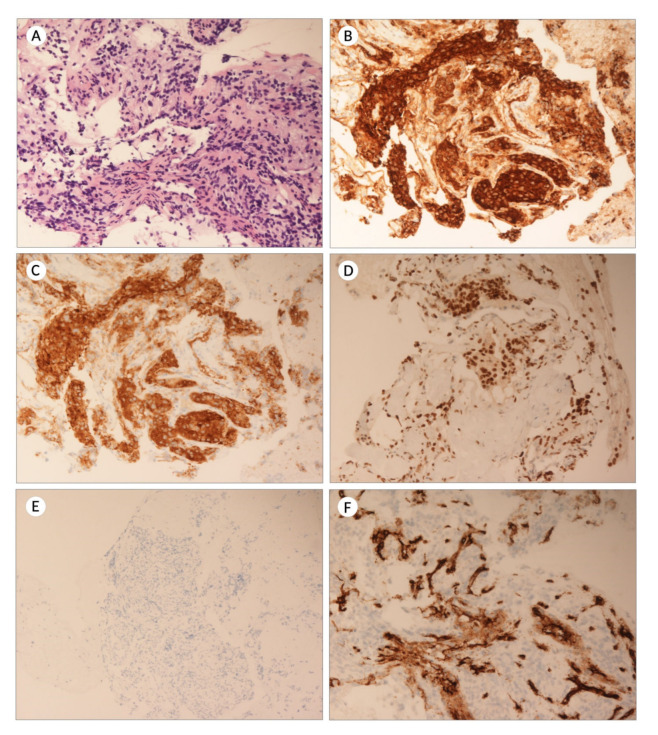
Representative images of a paraduodenal paraganglioma from our practice diagnosed with EUS-FNB, with a combination of H&E histomorphology and immunohistochemistry. (**A**), H&E ×200; (**B**), Chromogranin ×200; (**C**), Synaptophysin ×200; (**D**), GATA-3 ×200; (**E**), Keratin ×100; and (**F**), CD34 ×400.

Microscopic examination of the FNB material revealed the presence of neoplastic cells, mostly arranged in syncytial groups (Figure 2). Immunohistochemistry was performed; the neoplastic cells were positive for chromogranin, synaptophysin, CD56, and GATA-3, whereas they were negative for Keratin, Melan-A, PAX8, and DOG-1. CD34 staining revealed the rich network of transgressing blood vessels, while S100 the population of spindle, “sustentacular” cells within the neoplasm. Preoperative diagnosis was paraganglioma.

The mass was subsequently excised and surgical pathology confirmed the abovementioned preoperative diagnosis. Immunohistochemistry against SDHA, SDHB, and ATRX was additionally performed, and the neoplastic cells within the mass were found to retain their staining.

## 4. Discussion

To our knowledge, this is the first literature review summarizing the published data of abdominal extra-adrenal PPGLs diagnosed with FNA and/or FNB. This study showed that PPGLs preoperative diagnosis using the abovementioned modalities is feasible, especially when pathologists are familiar with the cytomorphologic features and immunochemistry is additionally applied on the cytologic or histologic material. As Table 1 shows, the main reason for misdiagnosis was the absence of a complete immunochemical panel. Possible reasons for this could be either that the physicians involved did not suspect a PPGL diagnosis or the pathologic material was inadequate for ancillary studies. The vast majority of the 36 included paragangliomas were retroperitoneal and most appeared in females (67%), in accordance with the literature [25], while FNA (31/36 cases) was more often used to sample them compared to FNB. In 2/36 patients, transient hypertension was reported during the EUS-FNA procedure [16,17]. Where as a few of these cases were symptomatic at diagnosis (e.g., presence of abdominal pain), they often appeared in asymptomatic patients, being detected incidentally with radiology (Table 1). Most patients exhibited a favorable clinical behavior, yet 5/36 patients (13.9%) exhibited a recurrence or widespread disease and one patient died 48 months following her diagnosis.

According to the literature, abdominal extra-adrenal paragangliomas are most often found in the periaortic and pericaval regions [34,35]. Notably, our analysis revealed that, when focusing on the subgroup undergoing biopsy with FNA or FNB, extra-adrenal PPGLs were most often reported to be located around and/or within the pancreas (21/36; 58.33%). In such cases, the first impression of clinicians is a more common primary pancreatic neoplasm rather than the rare paraganglioma, especially in asymptomatic patients devoid of clinical presentation related to the hypersecretion of catecholamines [4,14,25]. Of the pancreatic neoplasms, the most likely differential was found to be an epithelial NET (Table 1). Both PanNETs and paragangliomas are neuroendocrine neoplasms exhibiting similar morphologic characteristics, such as the presence of loosely clustered and isolated epithelioid or polygonal cells, various degrees of pleomorphism, granular cytoplasm, and nuclei with stippled, “neuroendocrine-like” chromatin. In addition, both PanNETs and PPGLs could contain binucleated or even multinucleated giant cells, and both are positive for neuroendocrine markers with immunochemistry, including chromogranin and synaptophysin. In contrast to the PanNETs though, paragangliomas are negative for Keratins (e.g., AE1/AE3 or CAM5.2) or CEA with immunochemistry (as they are non-epithelial neoplasms), whereas they exhibit immunopositivity for GATA-3 and tyrosine hydroxylase (TH) [5,36,37,38,39]. Additionally, in contrast to the peripancreatic paragangliomas, PanNETs are typically located inside the pancreatic parenchyma. However, this is not always straightforward during the radiologic or endoscopic examination, and neoplasm initially thought to be intra-pancreatic are subsequently found to be extra-pancreatic during surgery [4,5,32]. Apart from epithelial NETs, other erroneous preoperative interpretations of peripancreatic paragangliomas revealed in our study included the diagnoses of spindle cell neoplasm, pancreatic pseudocyst, pancreatic adenocarcinoma, or anaplastic carcinoma, particularly when immunochemistry was not performed [4,32].

As shown in our study, neoplasms other than epithelial NETs could also enter the main differential diagnosis, depending on the retroperitoneal location of extra-adrenal PPGLs. For instance, in para-gastric or para-duodenal lesions, a gastrointestinal stromal tumor (GIST) needs to be ruled out; GISTs are positive for DOG1 and CD117 with immunochemistry whereas they are negative for neuroendocrine markers, in contrast to paragangliomas. Furthermore, when found in the perirenal space or the retroperitoneal soft tissue, a renal cell carcinoma or a soft tissue tumor, such as a schwannoma or sarcoma, should also be excluded. In addition, various metastatic carcinomas or a melanoma could often be considered as a potential diagnosis. Metastatic carcinomas would be Keratin positive, while melanomas positive for Melan-A, HMB-45, and diffusely positive for S100 with immunochemistry (in contrast to the paragangliomas, where only the sustentacular cells are highlighted with the S100 immunostaining) [14,40,41]. Lastly, when PPGLs are found in the head and neck area rather than the retroperitoneum, other lesions enter their differential diagnosis, for instance the medullary thyroid carcinoma [42].

The accurate diagnosis of a retroperitoneal PPGL is clinically important, especially its distinction from epithelial NETs. First, paragangliomas could be associated with a distinct clinical picture related to the activation of the sympathetic nervous system, while the metanephrines are regarded as biomarkers to monitor their response to therapy and potential recurrence. Furthermore, as paragangliomas often carry germline (more that 40% of the cases) and/or somatic mutations, genetic testing is offered to the affected patients and its results could impact patients’ management, follow-up, and prognosis [8,10,43,44]. For instance, the presence of SDHB mutations, ATRX mutations, and telomerase inactivation have been correlated with the neoplasm’s metastatic potential or multifocal primary disease [45,46]. Notably, the loss of SDHB expression detected with immunohistochemistry is considered a surrogate marker of a SDHB mutation, while PPGLs with the aforementioned immunohistochemical profile are classified as “SDH-deficient PPGLs”; similarly, the loss of SDHA expression implies SDHA-related disease, while a VHL-related pathogenesis could be featured by using the carbonic anhydrase IX (CAIX) immunostaining [1,3,47,48,49,50]. The latest WHO classification emphasizes that paragangliomas should not be classified as benign or malignant, as all of them have a metastatic potential, while there are no clear-cut criteria to foresee their behavior. In addition, this is not certain whether the presence of a new paraganglioma mass in a patient represents a metastasis or an asynchronous multifocal primary disease, especially in patients with germline susceptibility. Exceptions include the bones and lymph nodes, which do not normally contain chromaffin tissue elements, thus a paraganglioma found in these sites could be considered a metastasis [1,3,51]. To predict the metastatic potential of PPGLs, a few histopathologic scoring systems have been established, for instance the PASS and the GAPP systems [52,53].

This study has some important limitations. Data were extracted from case reports or small case series, which are generally considered of low-quality evidence. This could not be avoided though, as abdominal paragangliomas are rare lesions, while we additionally focused only on the ones diagnosed with FNA and/or FNB. Furthermore, we need to consider the reporting bias, as PPGLs suspected clinically as such were most likely not biopsied. Lastly, in six of the initially eligible studies reporting abdominal extra-adrenal paragangliomas, individual data could not be extracted; thus, these reports were excluded from the review (Figure 1).

## 5. Conclusions

This study showed that the preoperative diagnosis of abdominal extra-adrenal paragangliomas with FNA or FNB is feasible. Diagnostic pitfalls exist, but could significantly be avoided within a multidisciplinary setting and the application of immunochemistry on the cytologic or histologic material. The retroperitoneal paragangliomas most often aspirated with FNA or FNB were reported to be located around or within the pancreas, and a PanNET was the main differential diagnosis.

## Figures and Tables

**Table 1:** Literature review (2000–2022) of abdominal extra-adrenal paragangliomas diagnosed with FNA or FNB.

First Author, Year	Gender, Age	Location	Diameter (Radiology)	Clinical Presentation	Biopsy Type	Preoperative Diagnosis	Immunochemistry on FNA/FNB Material	Surgery Performed	Follow-Up
Radulovic, 2022 [19]	F, 48	Peripancreatic (Tail)	35 mm (EUS)	Incidental mass; diarrhea and metrorrhagia	EUS-FNA	PPGL	Chr (+), Syn (+), GATA-3 (+), S100 (+), Ker (−), Inhibin (−), PAX8 (−), WT-1 (−), Ki-67 (<3%)	Yes	No alterations found in subsequent genetic testing
Lanke, 2021 [5]	F, 73	Peripancreatic (Head)	19 mm (EUS)	Asymptomatic, incidental mass; metanephrine levels normal	EUS-FNA	PPGL	Chr (+), Syn (+), GATA-3 (+), Ker (−), Ki-67 (<1%)	No	Alive, no recurrence (12 mo)
Thakur, 2021 [20]	M, 58	Peripancreatic (Head and uncinate process)	78 mm (EUS)	Asymptomatic, incidental mass; metanephrine levels normal	EUS-FNA	PPGL	Chr (+), Syn (+), GATA-3 (+), Ker (−)	N/A	Under MIGB therapy, to be followed by surgery
Naito, 2021 [21]	F, 61	Peripancreatic (Head/greater omentum)	21 mm (CT)	Asymptomatic, incidental mass	EUS-FNA	NET	Chr (+), Syn (+), CD56 (+)	Yes	Alive, no recurrence (6 mo)
Abbasi, 2020 [22]	F, 61	Peripancreatic (Head)	64 mm (EUS)	Asymptomatic, incidental mass	EUS-FNA	PanNET	Chr (+), Syn (+)	Yes	Alive, no recurrence (12 mo)
Yang, 2019 [23]	F, 67	Peripancreatic (Head)	50 mm (CT)	Abdominal pain, weight loss, nausea, vomiting	EUS-FNA	PPGL	Chr (+), Syn (+), CD56 (+), Ker (−), Ki-67 (<1%)	No	N/A
Nguyen, 2018 [24]	F, 70	Peripancreatic (Tail)	58 mm (EUS)	Constipation, satiety	EUS-FNA	Suggestive of PPGL	Chr (+), Syn (+), GATA-3 (+), S100 (+), Ker (−), ER (−), CDX2 (−)	Yes	N/A
Fite, 2018 [14]	M, 55	Retroperitoneal	97 mm	Discomfort	FNA	Consistent with PPGL	N/A	Yes	Recurrence after 9 years (same location)
Fite, 2018 [14]	M, 35	Retroperitoneal	83 mm	na	FNA	PPGL	N/A	Yes	Widespread bone and lung metastatic lesions at 5-year follow-up
Fite, 2018 [14]	M, 40	Peripancreatic	51 mm	Pain and hematuria; plasma metanephrine levels high	FNA	Suggestive of PanNET	N/A	Yes	Alive, no recurrence
Fite, 2018 [14]	F, 23	Peripancreatic	70 mm	Tachycardia; plasma chromogranin A levels high	FNA	NET	N/A	Yes	Alive, no recurrence
Zeng, 2017 [25]	F, 58	Peripancreatic (Head)	65 mm (MRI)	Abdominal pain	EUS-FNA	PPGL vs. PanNET	Chr (+), Syn (+), CD117 (−)	Yes	N/A
Zeng, 2017 [25]	F, 53	Peripancreatic	25 mm (CT)	Pelvic pain	EUS-FNA	NET	Chr (+), Syn (+)	Yes	N/A
Tumuluru, 2016 [26]	F, 62	Peripancreatic (Body)	32 mm (EUS)	Asymptomatic; incidental mass	EUS-FNA	Atypical epithelial cells	N/A	Yes	Alive, no recurrence (18 mo)
Zhang, 2014 [18]	F, 50	Widespread (pancreatic head; multiple liver lesions)	60 mm (CT) for the peripancreatic lesion	Headache, palpitation, sweating, hypertension	FNA	Suggestive of PPGL	Chr (+), Syn (+)	Yes	Died (48 mo after diagnosis)
Handa, 2014 [27]	M, 32	Paraaortic	N/A	Headache	FNA under US guidance	PPGL	N/A	N/A	N/A
Handa, 2014 [27]	F, 50	Paraaortic	N/A	Abdominal mass	FNA under US guidance	PPGL	N/A	N/A	N/A
Moslemi, 2012 [15]	F, 55	Perirenal (Intraperitoneal)	150 mm (CT)	Abdominal pain, anorexia, weight loss	FNA under US guidance	Undifferentiated carcinoma	N/A	Yes	Alive, no recurrence (12 mo)
Ganc, 2012 [28]	F, 37	Peripancreatic (Head)	35 mm	Asymptomatic; incidental mass	EUS-FNA	NET	Chr (+), Syn (+)	Yes	N/A
Laforga, 2012 [29]	M, 85	Paragastric	N/A	Abdominal pain	EUS-FNA	N/A	N/A	Yes	Alive, no recurrence (22 mo)
Singhi, 2011 [4]	F, 61	Peripancreatic (Tail)	140 mm	Abdominal pain	EUS-FNA	Pseudocyst	N/A	Yes	Alive, no recurrence (140 mo)
Singhi, 2011 [4]	F, 52	Peripancreatic (Body)	140 mm	Abdominal pain	EUS-FNA and FNB	PPGL	N/A	No	Widespread metastatic lesions, DOD (34 mo)
Singhi, 2011 [4]	F, 54	Peripancreatic (Head)	65 mm	Abdominal pain	EUS-FNA and FNB	PPGL	N/A	Yes	Alive, no recurrence (8 mo)
Singhi, 2011 [4]	M, 40	Peripancreatic (Body)	51 mm	Asymptomatic; incidental mass in radiology	EUS-FNA	PanNET	N/A	Yes	Alive, no recurrence (4 mo)
Singhi, 2011 [4]	F, 78	Peripancreatic (Body)	170 mm	Abdominal pain	EUS-FNA	Spindle cell neoplasm	N/A	Yes	Alive, no recurrence (2 mo)
Singhi, 2011 [4]	M, 44	Peripancreatic (Head)	55 mm	Asymptomatic; incidental mass	EUS-FNA and FNB	PPGL	N/A	Yes	Alive, no recurrence (2 mo)
Sangster, 2010 [30]	M, 50	Peripancreatic (Head)	N/A	Abdominal pain; hypertension	FNA	Poorly differentiated carcinoma	N/A	No (a surgical biopsy was though performed, providing the final diagnosis)	Alive, no recurrence (37 mo)
Rangaswamy, 2010 [31]	M, 45	Perirenal	120 mm (CT)	Asymptomatic; incidental mass; hypertension (metanephrine levels high)	FNA under CT guidance	Suggestive of PPGL	N/A	Yes	N/A
Kubota, 2010 [16]	F, 58	Paraduodenal	70 mm (CT)	Asymptomatic; incidental mass; transient hypertension during the EUS-FNA procedure; metanephrine levels high	EUS-FNA	Suggestive of PPGL	N/A	Yes	N/A
Jiménez-Heffernan, 2006 [13]	F, 58	Retroperitoneal	N/A	N/A	FNA	NET	Chr (+)	Yes	N/A
Jiménez-Heffernan, 2006 [13]	M, 47	Retroperitoneal	N/A	N/A	FNA	NET	NP	Yes	N/A
Akdamar, 2004 [17]	F, 62	Paraduodenal	66 mm (EUS)	Abdominal pain; transient hypertension during the EUS-FNA procedure	EUS-FNA	Suggestive of a neoplasm	N/A	Yes	N/A
Gong, 2003 [32]	F, 69	Organ of Zuckerkandl	50 mm (CT)	Asymptomatic; incidental mass	FNA under CT guidance	Anaplastic carcinoma of the pancreas	NP	Yes	N/A
Gong, 2003 [32]	F, 74	Retroperitoneal soft tissue	N/A	Large abdominal mass	FNA under US guidance	Pancreatic adenocarcinoma	NP	Yes	Recurrent PPGL lesion in the liver (60 mo)
Absher, 2001 [33]	M, 52	Retrocrunal, paracaval, and paraaortic lesions; also, bone (rib, vertebral) lesions	70 mm (CT)	Chest wall and back pain	FNA and FNB	PPGL	Chr (+), vim (+), Ker (−), EMA (−), CEA (−), desmin (−)	N/A	Widespread metastatic bone lesions
Our case	F, 35	Paraduodenal	65 mm (EUS)	Asymptomatic; incidental mass	EUS-FNB	PPGL	Chr (+), Syn (+), CD56 (+) GATA-3 (+), S100(+), Ker (−), PAX8 (−), Melan A (−), DOG1 (−)	Yes	Alive, no recurrence (10 mo)

**Note**: The term “peripancreatic” is used to describe the location of a lesion found around and/or within the pancreas. **Abbreviations**: PPGL, pheochromocytoma and paraganglioma; NET; neuroendocrine tumor; PanNET; pancreatic neuroendocrine tumor; EUS, endoscopic ultrasound; Chr, chromogranin; Syn, synaptophysin; Ker, Keratin; N/A, information not available; NP; not performed; mo, months.

## Data Availability

Data are contained within the article.
